# Age at the onset of tobacco smoking in South Africa: a discrete-time survival analysis of the prognostic factors

**DOI:** 10.1186/s13690-020-00503-1

**Published:** 2020-12-02

**Authors:** Adeniyi Francis Fagbamigbe, Rachana Desai, Ronel Sewpaul, Ngianga-Bakwin Kandala, Derrick Sekgala, Priscilla Reddy

**Affiliations:** 1 Department of Epidemiology and Medical Statistics, College of Medicine, University of Ibadan, Ibadan, Nigeria; 2 Division of Population and Behavioural Sciences, School of Medicine, St Andrews University, Fife, UK; 3 Division of Health Sciences, Populations, Evidence and Technologies Group, University of Warwick, Coventry, UK; 4 SAMRC/Wits Developmental Pathways for Health Research Unit, Faculty of Health Sciences, University of the Witwatersrand, Johannesburg, South Africa; 5 Health & Wellbeing, Human and Social Capabilities Division, Human Sciences Research Council (HSRC), 116 - 118 Merchant House, Buitengracht Street, Cape Town, 8001 South Africa; 6 Division of Epidemiology and Biostatistics, University of the Witwatersrand, School of Public Health, Johannesburg, South Africa; 7 Nelson Mandela University, Port Elizabeth, South Africa

**Keywords:** Tobacco smoking, South Africa, Birth cohorts, Sex, Race, Tobacco advertisement

## Abstract

**Background:**

While knowledge of onset of smoking tobacco, and associated risk factors can aid the formulation of evidence-based policy and interventions, such information is scarce in South Africa. We assessed age at onset of tobacco smoking in South Africa and identified its risk factors.

**Methods:**

We analysed data of 15,316 respondents aged 15–98 years from the 2012 South African National Health and Nutrition Examination Survey. Descriptive statistics and survival analysis techniques were used alongside weighted percentages.

**Results:**

Overall lifetime prevalence of smoking was 20.5%. Among the 3360 ever-smoked respondents, the overall median age at smoking onset was 18 years (Inter-quartile range (IQR) =5) with 2% starting before age 10 while 60% had smoked before age 20. Likelihood of tobacco smoking was higher among adolescents (<=20 years) and those aged 20–29 years than those aged > = 60 years, thrice higher among males, 29% higher among urban dwellers and thrice higher in Western Cape and Free State than in North West Province. The onset of tobacco smoking was earlier among males, wealthier and “coloured” people from Northern and Eastern Capes.

**Conclusion:**

The onset of tobacco smoking peaked at 15–22 years and varied by province, sex, location, race and other characteristics. The age restrictions on smoking in South Africa has changed over time, coupled with the recent open and electronic advertisement of tobacco, and social media could have influenced the earlier onset of tobacco smoking in South Africa. Stricter regulations on tobacco-related advertisement and sales should be implemented.

## Background

Tobacco use is responsible for over 8 million deaths every year and this figure is projected to rise to approximately 10 million deaths per year by 2030 [1] and has been ranked as one of the leading causes of preventable deaths globally [2]. According to the WHO, the average global tobacco smoking among populations aged 15 years and older was 21% [3]. Moreover, South Africans aged 15 years and older reported past month tobacco smoking as high as 31.4% [4]. Tobacco use is a risk factor to a range of disability and diseases such as lung cancer, stroke, heart disease and chronic respiratory disease [5, 6].

An important determinant of smoking behaviours in later life is the age of smoking onset. Research across the globe has shown that the majority of adults initiated tobacco use before the age of 18 [7]. In developing countries, including South Africa, the average age of tobacco initiation was between 12 and 19 years [8–11]. Studies have shown that those who initiate smoking at a young age have greater nicotine dependence than those initiating later [12, 13]. Early age of smoking onset has furthermore been associated with a likelihood of regular smoking, placing individuals at higher risk for tobacco-related mortality and morbidity [9, 14]. If the age of smoking onset continues to decrease, tobacco use will result in the death of 250 million children and young people alive today, many of them in developing countries [15, 16]. Therefore, a better understanding of the age of when people initiate smoking, as well as when they started smoking is essential to develop more effective prevention efforts and smoking cessation programs for those who are most at risk.

A substantial body of research has identified a broad range of determinants for tobacco use at the individual and the environmental level. Determinants such as demographics (age, gender, race, location), socioeconomic status (SES) (education, occupation and income), psychosocial (norms, attitudes, self-efficacy, knowledge) the social network and policy, have all been linked to the smoking onset and progression [2, 5, 7, 9, 11, 12, 15–21]. In a recent study, Morojele et al. found that 44% of South African adolescents reported lifetime tobacco use. The authors controlled for the adolescents’ age, gender, ethnicity and SES in assessing shared and unshared risks of tobacco use among South Africa adolescents, evidence of the significance of these factors [22]. Males have been reported to have a higher prevalence of smoking than the females in South Africa [11]. Also, educational attainment, household wealth quintiles, employment status and location of residence as measures of deprivation have been reported to be associated with smoking [23]. Lau et al. found a significant non-linear association between deprivation and smoking prevalence [23]. The authors reported that the smoking prevalence ratio was highest among those in the middle range but lowest at the uppermost and lowest ends of the deprivation index. Similarly, smoking has been reported to differ by ethnic groups and races in the country. In South Africa, the “coloured” population have been reported to have the highest prevalence of smoking [11, 23, 24].

However, information on the age of smoking onset in low and middle-income countries has been seriously limited by the lack of population-based studies using a standardized methodology [25]. Moreover, research on tobacco use usually report the prevalence of smoking alone, and only a few studies in South Africa provide data on the incidence of smoking onset and factors associated with the timing of the onset. The current study aims to gain an understanding of the age at onset of smoking, the cumulative risk of smoking, and the associated risk factors using a national sample of South African adults.

## Materials and methods

We used the data from the 2012 South African National Health and Nutrition Examination Survey (SANHANES) from individuals aged 15 and older [26].

### Study population

The first round of the SANHANES (SANHANES-1) included a sample of all individuals living in South Africa. All persons living in occupied households were eligible to participate. It excluded individuals staying in educational institutions, old-age homes, hospitals, homeless people, and uniformed-service barracks.

### Study design

The SANHANES-1 obtained questionnaire-based data through interviews in combination with health measurements obtained through clinical examination, a selection of clinical tests as well as the collection of a blood sample. Questions on tobacco use behaviours were administered to individuals aged > = 15 years. SANHANES-1 was a cross-sectional survey providing baseline data for future longitudinal analysis.

### Sampling

The survey applied a multi-stage disproportionate, stratified cluster sampling approach. A master sample of 1000 census enumeration areas (EAs) from the 2001 population census from a database of 86,000 EAs was mapped in 2007 using aerial photography. The selection of EAs was stratified by province and locality type. In the formal urban areas, the race was also used as a third stratification variable (based on the predominant race group in the selected EA at the time of the 2001 census). The allocation of EAs to different stratification categories was disproportionate. Based on the Human Sciences Research Council (HSRC) 2007 Master Sample, 500 EAs representative of the socio-demographic profile of South Africa were identified and a random sample of 20 visiting points was randomly selected from each EA, yielding an overall sample of 10,000 households. All members within a sampled household were eligible to participate. The final sample consisted of 8166 valid households, with 27,580 eligible individuals. A total of 25,532 (92.6%) individuals participated in one or more of the interviews, physical examination and clinical examination, of which 15,316 were aged 15 years or older and answered tobacco-use related questions. Further details on sample procedures are described in Shisana et al. [26].

### Variables

The outcome variable is the age of onset of tobacco smoking (TS). Respondents were asked if they have ever smoked tobacco. Those who answered in affirmative were then asked the age when they started smoking or using tobacco. The independent variables, selected based on existing literature [2, 5, 7, 9, 12, 15–21] are age groups (< 20, 20–29, 30–39, 40–49, 50–59 and > =60), Sex (Male, Female), Location (Rural, Urban), Educational Attainment (No formal education, Grade 8–12, Higher education), Province (Western Cape, Eastern Cape, Northern Cape, Free State, KwaZulu Natal, North West, Gauteng, Mpumalanga, Limpopo), Race (African, White, “Coloured”, Indian and Other), Nationality (South African Citizen, Non - Citizen (Permanent Residence), Non-Citizen (Refugee) and Others), Marital Status (Never Married, Currently Married and Formerly Married), Employment Status (Unemployed, Employed) and Wealth Quintile (poorest, poorer, middle, richer and richest). These variables have been identified in previous studies to be associated with tobacco smoking.

### Statistical analysis

The analytic sample consisted of 15,316 individuals aged 15 years and older who responded to questions on tobacco use as sensitive questions such as tobacco-use were only asked from respondents of that age group. Descriptive statistics and survival analysis techniques were used to analyse the data. Percentages were weighted using sampling weights. Kaplan Meier estimates were generated to describe time to the onset of smoking using survival functions. Log-rank tests were used to test the equality of survival curves at 5% significance level. In the multivariate analysis, a survival analysis regression model was used to model the age at the onset of TS among the studied population. A survival regression model is used in follow-up studies to determine prognostic factors of the timing of an event of interest which is the age at the onset of TS in this study. These models work by combining two variables as the dependent variable. The two variables are (i) censoring indicator (ever smoked and never smoked in the current study) and (ii) survival time (measured as the age at the onset of smoking for the ever-smoked individuals and as the current age as of the date of data collection for the never-smoked individuals). We used the Cox proportional hazard model, one of the survival regression models, to determine the independent variables that predicted the age at the onset of TS in South Africa. The Cox models are based on the assumption of the proportionality of the hazards. The validity of this assumption was assessed using the log-log transformation of the Kaplan-Meier survival curves. The variables that were significant at *p* = 0.10 in the bivariate Cox regression models were identified as candidates and added to the multiple Cox regression model to assess their contribution to the timings controlling for confounders. Hazard ratios and their associated 95% confidence intervals from the Cox regression analysis were evaluated to determine the association of the risk factors with the timing of the onset of tobacco use. The outcomes of the model are the hazard ratios and their associated 95% confidence intervals. The significance of the HRs was determined using a 95% confidence interval and Wald’s χ^2^ statistics. We verified whether the proportional-hazards assumption was violated or not across all levels of respondent characteristics. Data were weighted to adjust for unequal population sizes across the EAs and the provinces. All analysis was conducted using Stata version 14 (StataCorp, Texas, USA 2016).

The population at risk in this study comprised every individual involved in the study since any person can start smoking at any point in their lifetime. The duration from the date of birth to age at the onset of tobacco smoking, ‘T’, is assumed to be a discrete random variable that is always positive. The observation continues from birth until time ‘t’, at which the event of interest (age at the onset of TS) occurs. The study ends for an individual at a time ‘T = t’ if he/she started smoking, else such an individual is “censored” as at the survey date. We computed the survival time as the age at onset of smoking for those who had ever smoked or started on the survey day while, for those who have never smoked, the survival time is their age on the survey date. The censoring index for the ever-smoked respondents is “1” while it is “0” for those who have never smoked. We accounted for the time-varying effects of the age of the respondents, household wealth quintile and educational attainment on the timing of smoking onset among the respondents.

## Results

Among the 15,316 respondents, 22.9% were aged 19 years or less while 8.7% were 60 years or more. Also, 54.2% were females, 63.6% lived in urban areas, 24.5% from Gauteng and 2.3% from North Cape province, 77.9% are Africans while 10.1% classified themselves as White, as seen in Table 1. Overall, the lifetime prevalence of smoking, that is, having ever smoked tobacco, in this population was 20.5%. The lifetime prevalence of smoking (10.7%) was lowest among those aged 19 years or less, while those aged 50 to 59 years had the highest lifetime prevalence (29.2%). The prevalence was also high among males (33.2%), and those who classified themselves as coloured (45.9%). In terms of location, formal urban and rural areas reported lifetime rates of 23. 7 and 25.6% respectively. The Western Cape (37.9%) reported the highest lifetime prevalence of smoking, followed by the Northern Cape (33.2%) and Free State (30.7%). All other background characteristics of the respondents showed consistent lifetime smoking rates (Table 1).

**Table 1:** Distribution of respondents and the lifetime prevalence of tobacco smoking

Characteristics	%	*n* = 15,316	Lifetime prevalence of smoking %(95% CI)
Age in years			
< 20	23.6	3607	10.7 [9.0–12.5]
20–29	21.3	3258	21.0 [18.9–23.3]
30–39	16.4	2506	23.8 [21.4–26.4]
40–49	15.4	2366	27.2 [24.7–29.9]
50–59	12.2	1870	29.2 [26.1–32.4]
> =60	11.2	1709	20.1 [16.5–24.1]
Sex			
Males	41.6	6337	33.2 [31.1–35.5]
Females	58.4	8897	10.2 [8.9–11.7]
Location			
Rural	33.5	5127	16.6 [14.8–18.6]
Urban	66.5	10,189	23.1 [21.4–25.0]
Geographic Location			
Urban Formal	54.1	8293	23.7 [21.7–25.9]
Urban Informal	12.4	1896	19.8 [16.7–23.3]
Rural Informal (Tribal)	21.3	3269	14 [12.2–16.0]
Rural Formal (Farms)	12.1	1858	25.6 [20.9–31.0]
Province			
Western Cape	14.0	2151	37.9 [32.9–43.2]
Eastern Cape	10.7	1637	21.6 [18.4–25.1]
Northern Cape	6.5	995	33.2 [25.7–41.7]
Free State	5.4	831	30.7 [26.9–34.9]
Kwazulu Natal	16.6	2547	20.2 [17.6–23.0]
North West	12.6	1932	14.5 [12.5–16.7]
Gauteng	17.2	2631	15.2 [12.8–18.1]
Mpumalanga	8.6	1324	17 [13.2–21.5]
Limpopo	8.3	1268	13.7 [10.2–18.1]
Race			
African	66.9	10,163	17 [15.8–18.3]
White	4.7	718	25.1 [20.3–30.7]
Coloured	19.8	3004	45.9 [42.7–49.1]
Indian	8.5	1295	27 [21.3–33.5]
Other	0.1	8	ISS
Nationality			
South African Citizen	98.5	14,880	20.8 [19.6–22.2]
Non - Citizen (PR)	1.0	148	21.4 [13.2–32.9]
Non-Citizen (Refugee)	0.4	57	18.4 [8.2–36.6]
Other (Specify)	0.1	16	ISS
Marital Status			
Never Married	45.3	5722	19.7 [18.1–21.4]
Currently Married	44.4	5616	23.9 [21.7–26.2]
Formerly Married	10.3	1300	21.4 [17.5–25.8]
Employment Status			
Unemployed	62.2	7736	20.5 [19.0–22.1]
Employed	37.8	4703	23.8 [21.8–26.0]
Educational Attainment			
No formal education	26.6	4003	24.5 [22.2–27.0]
Grade 8–12	63.6	9556	19.9 [18.4–21.5]
Higher education	9.8	1471	18.6 [15.3–22.3]
Wealth Quintile			
Lowest	20.4	2625	21.1 [18.5–23.9]
Lower	18.0	2309	17.3 [15.2–19.5]
Middle	18.9	2423	19.5 [17.0–22.2]
Higher	21.5	2764	23.8 [20.8–27.1]
Highest	21.2	2719	20.6 [18.0–23.5]
Total		15,316	20.5 [19.1–21.9]

*ISS* Insufficient Sample Size

A detailed analysis of ever-smoked respondents and their ages at the onset of smoking is shown in Table 2. Among the 3360 respondents who had ever smoked, the overall median age at onset of smoking was 18 years (Inter-quartile range (IQR) = 5) with 1.5% having started smoking before age 10 years. Concerning Province, 3.8% had started TS before attaining age 10 years in Limpopo compared with 0.1% in Gauteng, 74.9% started TS between ages 10 and 19 years in the Western Cape compared with 47.8% in the Free State. The median age at onset of smoking was 17 years (IQR = 4) in the Western Cape compared with 20 years (IQR = 6) in Free State.

**Table 2:** Distribution of age at onset of smoking and incidence rate of smoking among ever-smokers

Characteristics	n	Age at Onset of Tobacco Smoking(*n* = 3360)					
		< 10 year	10–19 year	20–29 year	After 30 year	Median	IQR
Age in years							
< 20	450	2 [0.9–4.6]	92.1 [87.4–95.1]	5.9 [3.3–10.4]	0.0 [0.0–0.0]	16	3
20–29	743	2.1 [1.1–4.0]	66.4 [61.7–70.8]	31.0 [26.8–35.7]	0.5 [0.1–1.7]	18	4
30–39	595	1.7 [0.8–3.9]	56.7 [50.3–63.0]	35.3 [29.5–41.6]	6.2 [4.0–9.4]	18	5
40–49	670	0.5 [0.2–1.3]	47.5 [42.2–52.9]	38.4 [33.4–43.6]	13.7 [9.4–19.4]	20	8
50–59	557	1 [0.4–2.3]	48.3 [40.7–55.9]	33.5 [27.6–39.9]	17.3 [13.4–22.0]	20	8
> =60	331	1.5 [0.5–4.6]	39.4 [29.5–50.2]	31.9 [23.8–41.4]	27.2 [16.9–40.7]	20	13
Sex							
Males	2218	1.7 [1.0–2.7]	59.1 [56.1–62.1]	33.0 [30.2–35.8]	6.2 [5.1–7.6]	18	5
Females	1113	1.1 [0.6–2.0]	59.0 [53.2–64.6]	24.6 [20.6–29.1]	15.4 [10.8–21.4]	18	6
Location							
Rural	895	1.9 [1.1–3.5]	54.7 [50.0–59.3]	34.2 [30.1–38.6]	9.2 [7.2–11.6]	18	6
Urban	2451	1.3 [0.8–2.2]	60.9 [57.5–64.2]	29.4 [26.5–32.5]	8.4 [6.4–11.0]	18	5
Geographic Location							
Urban Formal	2084	1.3 [0.7–2.3]	60.9 [57.0–64.6]	29.5 [26.2–32.9]	8.4 [6.1–11.3]	18	5
Urban Informal	367	1.5 [0.6–3.5]	60.9 [55.2–66.3]	29.1 [23.7–35.1]	8.6 [5.8–12.4]	18	5
Rural Informal (Tribal)	418	2.2 [1.0–4.6]	52.1 [46.7–57.5]	35.8 [30.7–41.4]	9.9 [7.4–13.0]	18	7
Rural Formal (Farms)	477	1.5 [0.7–3.6]	59.4 [50.7–67.6]	31.2 [24.7–38.6]	7.8 [5.0–11.9]	18	5
Province							
Western Cape	897	2.1 [1.3–3.5]	74.9 [69.3–79.8]	19 [15.6–23.0]	3.9 [2.2–6.7]	17	4
Eastern Cape	400	1.2 [0.3–4.0]	49.7 [43.7–55.7]	36.9 [31.6–42.5]	12.3 [9.0–16.5]	19	7
Northern Cape	356	1.6 [0.3–7.9]	64.1 [55.3–72.1]	27.7 [20.2–36.7]	6.6 [3.7–11.5]	18	4
Free State	249	0.9 [0.2–3.9]	47.8 [41.0–54.7]	42.8 [35.1–50.8]	8.5 [5.5–13.0]	20	6
Kwazulu Natal	472	2.4 [0.9–6.2]	59.3 [51.5–66.6]	27.7 [21.9–34.3]	10.6 [5.6–19.4]	18	5
North West	238	0.2 [0.0–1.7]	51.8 [44.6–58.9]	35.7 [29.5–42.4]	12.3 [8.5–17.6]	19	5
Gauteng	380	0.1 [0.0–0.8]	55.3 [48.4–62.0]	35 [27.8–42.9]	9.6 [5.7–15.7]	19	6
Mpumalanga	188	0.4 [0.1–2.5]	51.3 [43.3–59.3]	38.9 [31.8–46.5]	9.4 [6.6–13.2]	19	7
Limpopo	166	3.8 [1.6–8.5]	54.9 [43.0–66.3]	33.7 [24.5–44.3]	7.6 [4.3–13.3]	18	7
Race							
African	1591	1.8 [1.1–2.9]	52.0 [48.8–55.2]	36.1 [33.2–39.1]	10.1 [8.6–11.8]	19	6
White	177	0.8 [0.3–2.7]	65.7 [53.1–76.4]	21.2 [13.1–32.5]	12.3 [4.9–27.5]	18	4
Coloured	1285	1.3 [0.6–2.5]	76.4 [72.8–79.6]	19.8 [16.9–23.2]	2.5 [1.7–3.8]	17	4
Indian	270	0.3 [0.1–1.4]	61.1 [54.0–67.8]	31.9 [23.9–41.1]	6.6 [3.7–11.6]	18	4
Other	1	ISS	ISS	ISS	ISS	ISS	ISS
Nationality							
South African Citizen	3266	1.5 [1.0–2.3]	59 [56.3–61.7]	30.8 [28.4–33.2]	8.7 [7.1–10.6]	18	5
Non - Citizen (PR)	24	ISS	ISS	ISS	ISS	ISS	ISS
Non-Citizen (Refugee)	10	ISS	ISS	ISS	ISS	ISS	ISS
Other (Specify)	1	ISS	ISS	ISS	ISS	ISS	ISS
Marital Status							
Never Married	1183	2.0 [1.1–3.8]	64.5 [60.3–68.5]	27.7 [24.2–31.4]	5.8 [4.3–7.8]	18	5
Currently Married	1430	1.1 [0.6–1.8]	55.7 [51.3–59.9]	32.9 [29.1–36.9]	10.4 [8.0–13.4]	18	4
Formerly Married	281	0.3 [0.1–1.4]	41.3 [31.4–52.0]	36.5 [27.3–46.8]	21.9 [10.8–39.2]	20	4
Employment Status							
Unemployed	1663	1.6 [1.0–2.8]	60.2 [56.6–63.7]	29.6 [26.4–32.9]	8.6 [7.1–10.4]	18	5
Employed	1214	1.3 [0.7–2.4]	57.6 [53.3–61.8]	33.6 [29.8–37.6]	7.5 [5.3–10.7]	18	6
Educational Attainment							
No formal education	896	1.6 [0.8–3.5]	49.0 [44.0–54.0]	33.9 [29.5–38.5]	15.5 [12.6–18.9]	19	8
Grade 8–12	1758	1.5 [0.9–2.5]	62.0 [58.2–65.6]	29.8 [26.6–33.1]	6.8 [4.6–9.8]	18	4
Higher education	219	0.5 [0.1–2.9]	62.7 [51.5–72.7]	30.0 [21.5–40.1]	6.8 [2.2–19.1]	18	4
Wealth Quintile							
Lowest	558	1.3 [0.6–2.8]	50.8 [45.4–56.2]	37 [31.9–42.3]	10.9 [8.1–14.6]	19	7
Lower	462	5.1 [2.5–10.1]	52.8 [46.7–58.9]	30.9 [25.5–36.9]	11.2 [7.7–15.9]	18	7
Middle	506	1.6 [0.7–3.9]	60.9 [54.1–67.4]	30.2 [24.1–37.2]	7.2 [4.7–10.8]	18	5
Higher	716	1 [0.4–2.4]	61.6 [55.4–67.5]	31 [25.9–36.7]	6.4 [4.7–8.7]	18	4
Highest	560	0.6 [0.3–1.6]	64.8 [57.0–71.9]	26.9 [20.6–34.3]	7.7 [3.2–17.3]	18	4
Total	3360	1.5 [1.0–2.2]	59.1 [56.4–61.7]	30.8 [28.4–33.3]	8.6 [7.0–10.5]	18	5

*ISS* Insufficient Sample Size, *IQR* Interquartile range

**Fig. 1:**
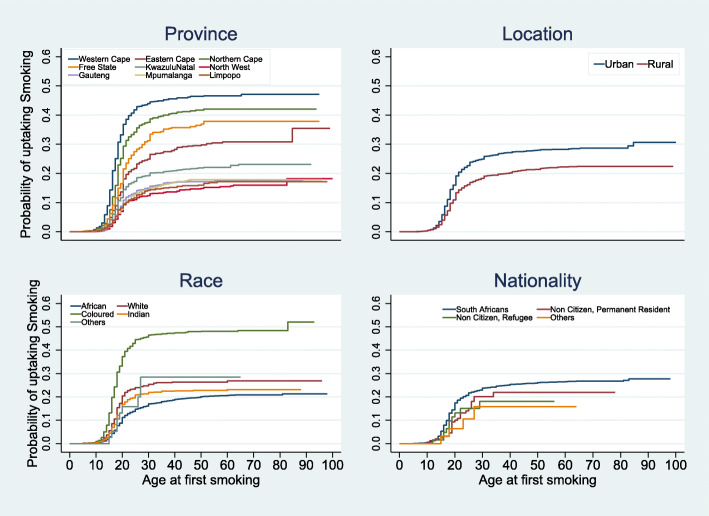
Survival functions of time to onset of smoking by the race, nationality, province and location of respondents

**Fig. 2:**
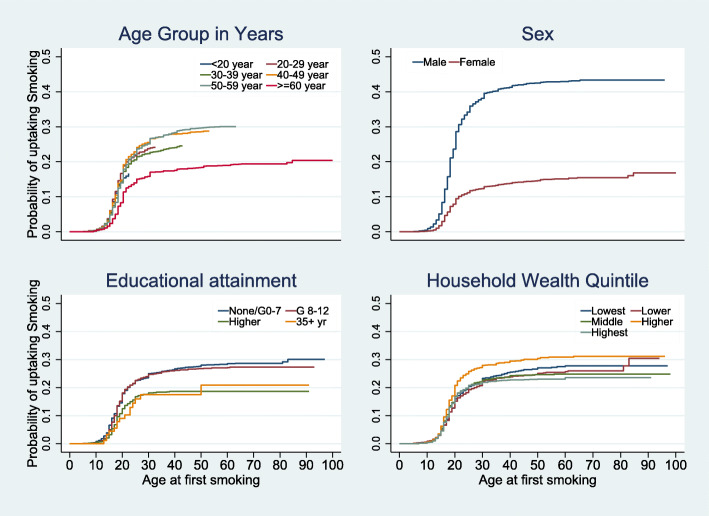
Survival functions of time to onset of smoking by age, sex, educational attainment and household wealth quintile of the respondents

Censoring the respondents who have never smoked as their current age as of the time of data collection, the survival curves of the onset of TS by selected characteristics are presented in Figs. 1 and 2. The Log-rank test for equality of survival curves showed that the curves were significantly different among the respondents’ province, race location, nationality, sex, birth cohort, and wealth quintile. All the curves showed a sharp increase in the probability of uptake between the ages of 15 and 22 years. The most noticeable differences were among the provinces, location, and sex. The probabilities were consistently highest in the Western Cape, Northern Cape and Free State. It was also higher in urban areas than in rural areas, and among Africans and South African nationalities, people from households in the highest wealth quintiles, younger persons, and males.

We computed the incidence rate (IR) of TS per 10,000 people per year in Table 3. For instance, the incidence rate of TS among all respondents was 65 per 1000. The IR was 31 among persons born aged 6 years or more, and 91 among persons aged 20 to 29 years, 120 among males and 34 among females, 79 among persons from households in higher wealth quintiles compared to those in lowest wealth quintiles. The IR was 148 among the coloured people compared to 46 among Africans, 145 in Western Cape, 115 in Northern Cape and 34 in the North West province.

**Table 3:** Adjusted and unadjusted determinants of timing of onset of smoking

Characteristics	IR per 10,000	HR(95% CI)	*p*-value	aHR(95% CI)	*p*-value
Age in years					
< 20	70	1.42 [1.23–1.64]^*^	0.000	1.27 [1.03–1.57]^*^	0.027
20–29	91	1.53 [1.34–1.75]^*^	0.000	1.42 [1.17–1.71]^*^	0.000
30–39	72	1.41 [1.23–1.62]^*^	0.000	1.27 [1.05–1.53]^*^	0.012
40–49	72	1.64 [1.43–1.87]^*^	0.000	1.43 [1.20–1.71]^*^	0.000
50–59	64	1.67 [1.46–1.92]^*^	0.000	1.46 [1.22–1.74]^*^	0.000
> =60	31	Reference			
Sex					
Males	120	3.40 [3.17–3.66]^*^	0.000	3.37 [3.08–3.68]^*^	0.000
Females	34	Reference			
Location					
Rural	63	Reference			
Urban	75	1.40 [1.30–1.51]^*^	0.000	1.29 [1.16–1.44]^*^	0.000
Province					
Western Cape	145	4.27 [3.70–4.93]^*^	0.000	2.72 [2.16–3.42]^*^	0.000
Eastern Cape	73	2.12 [1.81–2.49]^*^	0.000	2.02 [1.60–2.54]^*^	0.000
Northern Cape	115	3.37 [2.86–3.97]^*^	0.000	2.28 [1.78–2.91]^*^	0.000
Free State	96	2.72 [2.27–3.25]^*^	0.000	3.09 [2.42–3.94]^*^	0.000
Kwazulu Natal	53	1.56 [1.34–1.83]^*^	0.000	1.80 [1.42–2.28]^*^	0.000
North West	34	Reference			
Gauteng	43	1.19 [1.01–1.39]^*^	0.040	1.28 [1.01–1.63]^*^	0.040
Mpumalanga	40	1.16 [0.96–1.41]	0.122	1.60 [1.23–2.08]^*^	0.000
Limpopo	37	1.10 [0.90–1.34]	0.356	1.59 [1.21–2.09]^*^	0.001
Race					
African	46	Reference			
White	64	1.52 [1.30–1.78]^*^	0.000	1.98 [1.61–2.43]^*^	0.000
Coloured	148	3.33 [3.09–3.58]^*^	0.000	2.70 [2.40–3.04]^*^	0.000
Indian	55	1.27 [1.12–1.45]^*^	0.000	1.56 [1.29–1.89]^*^	0.000
Other	84	1.49 [0.77–2.86]	0.235	1.32 [0.55–3.16]	0.537
Nationality					
South African Citizen	65	1.86 [0.70–4.95]	0.216		
Non - Citizen (PR)	57	1.40 [0.49–4.00]	0.530		
Non-Citizen (Refugee)	56	1.30 [0.41–4.14]	0.659		
Other (Specify)	44	Reference			
Marital Status					
Never Married	78	1.34 [1.17–1.53]^*^	0.000	1.05 [0.88–1.24]	0.595
Currently Married	65	1.29 [1.13–1.46]^*^	0.000	0.90 [0.78–1.05]	0.201
Formerly Married	42	Reference			
Employment Status					
Unemployed	64	Reference			
Employed	76	1.11 [1.03–1.20]^*^	0.005	0.86 [0.78–0.94]^*^	0.001
Educational Attainment					
No formal education	61	1.52 [1.31–1.76]^*^	0.000	1.02 [1.01–1.03]^*^	0.000
Grade 8–12	72	1.47 [1.28–1.69]^*^	0.000	1.02 [1.01–1.02]^*^	0.000
Higher education	48	Reference			
Wealth Quintile					
Lowest	66	Reference			
Lower	60	0.94 [0.83–1.07]	0.349	0.90 [0.79–1.03]	0.140
Middle	62	0.98 [0.87–1.10]	0.691	0.78 [0.68–0.89]^*^	0.000
Higher	79	1.23 [1.10–1.38]^*^	0.000	0.77 [0.68–0.88]^*^	0.000
Highest	79	0.92 [0.82–1.03]	0.165	0.54 [0.46–0.63]^*^	0.000
Total	65				

*IR* Incidence rate *Significant at 5%

The crude hazard ratio (HR) and adjusted hazard ratio of factors associated with the onset of smoking are presented in Table 3. The test of the assumption of proportionality showed that the assumptions were not violated. Hence, the Cox model was appropriate to model the data. The “hazard” or “risk” of TS was higher among younger people than older people. For instance, the hazard was 42% (adjusted Hazard Ratio (aHR) = 1.42; 95% Confidence Interval (CI):1.17–1.71) higher among people aged 20 to 29 years than those aged 60 years or more. The adjusted hazard was three times (aHR = 3.37; 95% CI:3.08–3.68) higher among the males than the females and 29% times (aHR = 1.29; 95% CI:1.16–1.44) higher among urban dwellers than rural dwellers. The hazard of TS was about three times higher in both Western Cape (aHR = 2.72; 95% CI:2.16–3.42) and Free State (aHR = 2.42; 95% CI:3.94) and two times higher in Eastern Cape (aHR = 2.02; 95% CI:1.60–2.54) and Northern Cape (aHR = 2.28; 95% CI:1.78–2.91) than in the North-West Province. Other significant predictors are race, employment status and household wealth quintile.

## Discussion

The main goal of this paper was to explore the age at the onset of tobacco smoking, as well as the determinants of the age of smoking onset using recent national data in South Africa. Results of this study confirm that one in five people in South Africa had used tobacco in their lifetime. Consistent with one of the latest data from the WHO, the average global tobacco smoking among populations aged 15 years and older was 21% [3]. The median age at onset of smoking was 18 years. This is in agreement with the reported 18.17 years among males and 18.33 years among females in South Africa by Vellios et al. [11]. Moreover, the highest lifetime prevalence of smoking was among males, those who classified themselves as “coloured” and those residing in the Western Cape. Similar rates of past-month cigarette smoking among adult South Africans with the same demographic characteristics were found in another recent national survey [23, 24, 27]. The “coloured” people in South African have a long history of a high prevalence of smoking. In 1977, 4 of every 5 “coloured” males and half of “coloured” females were smokers [24]. Children may take to smoking because their parents and grandparents smoke, so it becomes a norm and remains prevalent over time. Smoking is seen as a way of life among the “coloured”. Although the prevalence is reducing, it is generally higher among the “coloured” than what is obtainable among other races. Despite widespread knowledge of the harm caused by smoking, and concerted tobacco control efforts in the last decade [18], additional efforts, are needed to ensure continued reduction in smoking prevalence amongst the South African population.

Findings from this study show trends in smoking onset over time. Tobacco smoking started among some individuals as early as age 9 years old, and more than half of South Africans who had ever smoked initiated smoking between the ages 10–19 years. Overall, the highest risk of smoking onset usually occurred between ages 15 and 22 years across the entire population, with younger people more at risk of initiating tobacco smoking at a younger age compared to older birth cohorts. It appears that over time, people in South African have started smoking at younger ages despite substantial decreases in the prevalence of smoking in the last decades [6], these findings are worrisome. Moreover, the introduction of new tobacco products such as electronic nicotine delivery systems has the potential to also reverse the gains of decades of efforts and public health policy in South Africa, and decrease the age of smoking onset [28]. Interestingly, national studies conducted in high-income countries have shown an increase in smoking onset becoming more concentrated during young adulthood aged 18–21 years [29–32] with evidence of this found in the more recent birth cohorts [33].

This study furthermore found considerable variations in the risk of early smoking onset between sex, geographical location and the different racial groups of South Africa. The risk of early smoking onset was highest among coloured people, who comprise a high proportion of the Western Cape and Northern Cape populations. It was also higher in urban areas than in rural areas and among males compared to females. Similar findings have been reported elsewhere [2, 7, 19]. South Africa is a middle-income country with large wealth disparities, increasing proportions of the population living in urban areas and distinctive social-cultural differences [34].

### Strengths and limitations

The present study is not without its limitations. The choice in the explanatory variables in this study was restricted due to the data being secondary. Respondents were also made to recall events that occurred in the past without any means of validating the responses, resulting in possible recall bias. Notwithstanding these limitations, the results from this study provided a scope of the determinants of the age at smoking onset in South Africa using nationally representative data. A major strength of this study is the use of recent nationally representative data made our findings generalizable in South Africa.

## Conclusions

We found that age at smoking onset in South Africa is reducing with most smokers starting at age 15–24 years. We found wide differences in the age of smoking onset concerning respondents’ birth cohort, province, sex, race, wealth quintile, and employment status. The current level of age restrictions on smoking coupled with an open and electronic advertisement, including social media, of tobacco could have influenced the early onset of tobacco smoking in South Africa. The introduction of new tobacco products and electronic nicotine delivery systems might have encouraged earlier onset of smoking among youths in South Africa. There is a need for health promotion and education on the consequences of smoking starting at the primary school level. Furthermore, stricter regulations smoking advertisements and the restriction of sales of tobacco to the youth are essential.

### Policy implications

Stricter regulations around advertisement of tobacco products and restricting sales of tobacco to the youth need to be implemented. Findings from high-income countries are also important to consider when implementing prevention programmes in South Africa, to avoid a generational shift in tobacco initiation in South Africa, as well as to counter the ever-evolving strategies the tobacco industry uses to market its products. The identified prognostic factors associated with the onset of tobacco smoking in South Africa show that the policy, community, organizational, interpersonal and individual levels of behaviour need to be targeted to comprehensively address smoking onset and progression, to further improve and develop smoking prevention programmes [35].

## Data Availability

The data underlying the study are available from the Dryad repository at the following DOI: 10.5061/dryad.8p33r.

## Abbreviations

aHR: Adjusted Hazard Ratio
CI: Confidence Interval
EAs: Enumeration areas
SANHANES-1: First round of the SANHANES
HR: Hazard ratio
HSRC: Human Sciences Research Council
IR: Incidence rate
IQR: Inter-quartile range
REC: Research Ethics Committee
SES: Socioeconomic status
SANHANES: South African National Health and Nutrition Examination Survey
TS: Tobacco smoking
